# Therapeutic trends of priming mesenchymal stem cells: A bibliometric analysis

**DOI:** 10.1016/j.bbrep.2024.101708

**Published:** 2024-04-08

**Authors:** Kamal Hezam, Enze Fu, Jun Zhang, Zongjin Li

**Affiliations:** a Nankai University School of Medicine, Tianjin, 300071, China; b Tianjin Key Laboratory of Human Development and Reproductive Regulation, Tianjin Central Hospital of Gynecology Obstetrics, Nankai University Affiliated Hospital of Obstetrics and Gynecology, Tianjin, 300052, China; c Department of Anesthesiology and Pain Medical Center, Tianjin Union Medical Center, Nankai University, Tianjin, 300121, China; d National Key Laboratory of Kidney Diseases, Chinese PLA General Hospital, Beijing, 100853, China

**Keywords:** Mesenchymal stem cells, MSCs, Priming, Preconditioning, Bibliometric analysis

## Abstract

Mesenchymal stem cells (MSCs) have gained substantial attention in regenerative medicine due to their multilineage differentiation potential and immunomodulatory capabilities. MSCs have demonstrated therapeutic promise in numerous preclinical and clinical studies across a variety of diseases, including neurodegenerative disorders, cardiovascular diseases, and autoimmune conditions. Recently, priming MSCs has emerged as a novel strategy to enhance their therapeutic efficacy by preconditioning them for optimal survival and function in challenging in vivo environments. This study presented a comprehensive bibliometric analysis of research activity in the field of priming mesenchymal stem cells (MSCs) from 2003 to 2023. Utilizing a dataset of 585 documents, we explored research trends, leading authors and countries, productive journals, and frequently used keywords. We also explored priming strategies to augment the therapeutic efficacy of MSCs. Our findings show increasing research productivity with a peak in 2019, identified the United States as the leading contributor, and highlighted WANG JA as the most prolific author. The most published journal was Stem Cell Research & Therapy. Keyword analysis revealed core research areas emerging hotspots, while coword and cited sources visualizations elucidated the conceptual framework and key information sources. Further studies are crucial to advance the translation of primed MSCs from bench to bedside, potentially revolutionizing the landscape of regenerative medicine.

## Highlights

• Mesenchymal stem cells (MSCs) have emerged as promising candidates for regenerative medicine.
• Priming MSCs, a novel strategy, enhances their therapeutic efficacy for optimal in vivo survival and function.
• This bibliometric analysis provides a comprehensive overview of research activity in the field of priming MSCs.
• This review provides a valuable resource for researchers and clinicians interested in the field of primed MSCs.

## 1 Introduction

Mesenchymal stem cells (MSCs) are pluripotent stem cells found in adult organisms, and characterized by their self-renewal and ability to differentiate into multiple lineages. They can be sourced from a variety of tissues, including bone marrow, umbilical cord, placenta, peripheral blood, and amniotic fluid. Due to their inherent property of targeted homing to injured tissues, MSCs open avenues for targeted therapeutic applications [1,2]. MSCs are known to secrete a range of paracrine factors instrumental in tissue repair and immunomodulation. Their characterization is primarily based on the expression of distinctive cell surface markers such as CD105^+^, CD73^+^, CD90^+^, CD34^−^, CD45^−^, CD14^−^, CD79^−^and HLA-DR^−^ [[3], [4], [5]]. Notably, MSCs exhibit low immunogenicity due to the lack of MHC class I molecules and the costimulatory molecule CD40, alongside minimal expression of MHC class II molecules, reducing the risk of rejection after transplantation [6,7]. Therefore, MSCs are highly promising for cell therapy in treating various diseases across human and animal models, playing key roles in immunoregulation, anti-inflammatory responses, and tissue regeneration [8,9].

Numerous strategies have been developed to enhance the therapeutic efficacy of mesenchymal stem cells. Priming is one effective approach that has gained recognition for improving MSC therapeutic outcomes. Priming of MSCs can be accomplished with bioactive substances such as cytokines, growth factors, hormones, and vitamins, along with hypoxia, biomaterials, pharmacological agents, and chemical factors to augment their therapeutic potential by modulating their secretory behavior [[10], [11], [12]]. Prior investigations have explored the beneficial effects of MSC priming through various biofactors, including IFN-γ, TNF-α, IL-1α-β, FGF-2, LPS, IL-17A, TLR3 and IGF-1 [10,11,[13], [14], [15], [16], [17]]. The results of these studies indicate promising enhancements in the treatment profiles of MSCs for a variety of diseases. For instance, priming MSCs with TNF and IFN-γ has been found to assist in bone regeneration and immune modulation, in addition to promoting unique microRNA expression within MSCs and their exosomes. Additionally, priming MSCs with IFN-γ has been shown to escalate their expression of immunosuppressive molecules such as TGF-, HGF, and PGE2, which facilitate their immunosuppressive impact on activated lymphocytes [[18], [19], [20]]. Therefore, we highlight the importance of MSC priming as a therapeutic strategy with potential applicability across a broad spectrum of ailments.

Bibliometric analysis is a significant and advanced research technique widely used in health and other sciences. It employs statistical methods to reveal patterns, track trends, and identify relationships in scholarly materials like research papers, books, and other academic resources [21,22]. Similar to how biology functions within academic publishing, bibliometrics offers key insights into various domains by scrutinizing both published information and its associated metadata. This analytical process provides evaluative and relational methods instrumental in categorizing scholarly production, delineating the academic field, and monitoring evolving trends. These works assist researchers in identifying pertinent studies, assessing evidence, and amalgamating results to further knowledge within their respective fields [23,24]. Modern bibliometric analysis has expanded to incorporate text mining and visualization tools. These additions facilitate the capture of topic evolution and research communities and the identification of emerging trends, thereby providing a visual representation of research findings [25]. As a result, its use in medical and health sciences has significantly increased in recent years. However, there is no comprehensive, up-to-date bibliometric and visualization study on MSC priming and its therapeutic impacts. This research offers insight into the current status and future directions of the field, uncovering major themes, trending keywords, and research hotspots. It aids researchers in identifying gaps and directing future studies. It also plays a vital role in medical research by mapping the evolution of scientific fields and facilitating evidence-based decision-making [[26], [27], [28]]. One of the core values of our analysis lies in its ability to guide future research efforts strategically. By highlighting areas of intense research activity as well as emerging themes, we illuminate pathways that hold promise for significant breakthroughs. For researchers at all stages of their careers, this serves as a beacon, directing them towards contributions that could fill existing gaps or further innovative applications of MSC priming. This strategic insight is particularly beneficial in a rapidly evolving field, where the efficient allocation of time and resources is paramount for sustained progress.

## 2 Methods

### 2.1 Study design

This study employed a retrospective approach to conduct a comprehensive bibliometric analysis and visualization study. The primary aim was to examine a wide range of relevant literature and gather valuable insights into the subject matter. By utilizing this retrospective design, the researchers were able to delve into previously published work and identify notable trends, patterns, and key findings pertaining to the field of interest. This approach provided a robust foundation for the study's objectives and facilitated a thorough analysis of the research landscape surrounding the priming of MSCs.

### 2.2 Data source and search strategy

On July 03, 2023, a comprehensive search was conducted in the Web of Science Core Collection (WoSCC) database, focusing on scientific literature concerning the priming of MSCs. This process involved Boolean search methods using targeted keywords in the title field such as “Priming mesenchymal stem cells” OR “preconditioning mesenchymal stem cells” OR “preconditioned mesenchymal stem cells” OR “Primed mesenchymal stem cells”. The search was limited to documents published in English to ensure consistency and comprehension of our analysis. The primary search strategy extracted a total of 585 publications pertaining to these keywords. These raw data represented a broad collection of potentially relevant resources, requiring thorough examination to determine their significance and applicability to the research question at hand. To refine this corpus, an exhaustive review of the titles and abstracts of all located documents was performed. This critical step in the research process facilitated the elimination of extraneous or irrelevant materials and ensured that only the most pertinent documents were retained for further analysis.

### 2.3 Data extraction

The data extraction process was thorough, involving collecting various details from each scientific work. Initially, the title of the publication was noted for a summary and initial data categorization. Authors' names were cataloged to gain insight into field contributors, and collaborations, and to identify prolific researchers. In addition, the journal name indicated the research area and scientific community interest. Keywords associated with each publication provided a quick overview of the main topics, useful for further categorization or meta-analysis. Institutional affiliations indicated leading research centers, and the first author's country affiliation helped understand geographic research distribution. This could reveal leading countries and potential regional research focus differences. Furthermore, the total citation count for each publication was collected, serving as an estimate of the paper's influence and reach within the scientific community.

### 2.4 Data analysis

The collected data were exported to Microsoft Excel 2019 and a bibliographic information file (BIB file). The raw data were then represented in terms of frequencies and percentages. Several bibliometric measures, such as the annual number of publications, the most published authors, leading journals, most active institutions and countries, frequently employed author keywords, and the most highly cited articles were calculated and evaluated with the R software package to determine the impact of the published articles [29]. The influence and reach of published articles within the field of priming mesenchymal stem cells have been assessed using the R software package. This software facilitated the statistical analysis of bibliometric data, allowing us to compute metrics such as citation counts, authors' contributions, h-index, etc. Through these metrics, we could quantify the scientific contribution and visibility of the research articles, identifying those that have significantly shaped the discourse and development of MSC priming. The R software package, with its comprehensive set of tools for data analysis and visualization, enabled us to systematically evaluate the breadth and depth of the articles' influence across the scientific community. It was employed to perform further various analyses, such as visualization of the countries of corresponding authors, a word cloud analysis, and the calculation of the mean total citations per year. Network visualizations, such as coauthorship among countries, author keywords, and cited sources, were also created using VOSviewer software version 1.6.16 for Windows, available at http://www.vosviewer.com [30]. This software allowed for a deeper understanding of the relationships and interconnections within the collected data.

### 2.5 Ethics statement

The study did not involve the direct recruitment of human participants or the use of animals. Therefore, there was no need for ethical considerations or approvals.

## 3 Results

The annual quantity of scientific production on priming mesenchymal stem cells.

**Fig. 1:**
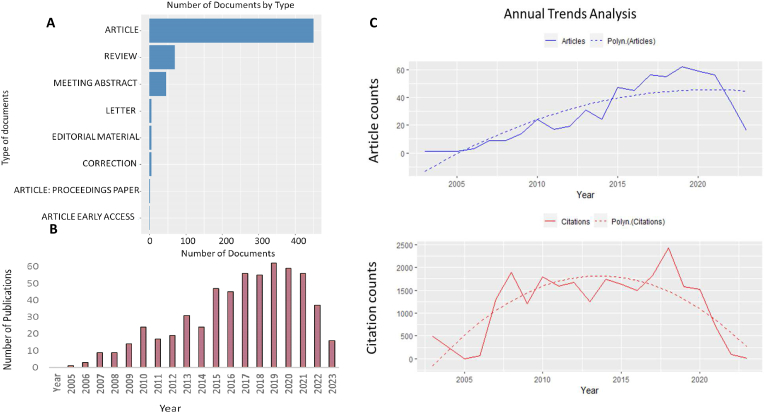
Scientific production on priming mesenchymal stem cells. A. Distribution of document types. B. Annual scientific production. C. Trends in annual article publications and citations with a polynomial (polyn) regression line (dashed) indicating the trend over time. The polynomial regression line models the relationship between time and the number of publications or citations, highlighting general trends.

This comprehensive investigation involved an analysis of 585 documents published in the English language, focusing on priming mesenchymal stem cells. The majority of these documents, a substantial number (451), took the form of original articles that presented new research findings or innovative theoretical perspectives. In addition, there were 70 review documents that provided synthesized examinations of existing knowledge (Fig. 1A).

The trends in annual publication activity for two decades, from 2003 to 2023, are visualized in (Fig. 1B). Notably, research productivity began to increase after 2003, peaking in 2019 with 62 documents published. This high level of activity was closely followed by the next year, 2020, which saw the publication of 59 documents. This upward trajectory contrasts sharply with the modest beginnings of this research area. The years 2005, 2006, and 2007 saw a relative scarcity of published documents, with their respective publication counts standing at 1, 3, and 9. As of mid-2023, 16 publications have been recorded, with early papers receiving the highest average number of citations (Fig. 1C). This figure only accounts for the first six months of 2023. If the current rate of publication continues, it is reasonable to expect that the total number of publications for 2023 will rise further in the remaining months, further contributing to the overall growth of research in the field of priming mesenchymal stem cells. In the analysis of the annual scientific production on priming mesenchymal stem cells, we observed a marked increase in publications, indicating growing interest and recognition of the potential of MSC priming in regenerative medicine and other related fields. The peak reflects heightened research activity, possibly driven by significant findings, technological advancements, or increased funding. The consistent output demonstrates ongoing interest and the continued relevance of MSC priming research. The alignment of these trends with the overall growth of scientific output across all sciences indicates that while MSC priming research is a niche area, it remains an integral part of the expanding landscape of biomedical research, contributing valuable insights and advancements.

### 3.1 Priming strategies to improve the therapeutic efficacy of mesenchymal stem cells

Various strategies have been proposed to improve the therapeutic potential of stem cells. The therapeutic effectiveness of MSCs was enhanced by priming them with biofactors and chemical factors, which controlled their secretion [10,12] (Table 2). Previous works have investigated the potential roles of MSC priming by a variety of biofactors, such as IFN-γ [13,31,32], TNF-α [14,33], IL-1α-β [15,34], FGF-2 [16], LPS [35], IL-17A [17], TLR3 [36], IGF-1 [37,38], IL-6 [39], IL-8 [40,41], IL-3 [42,43], IL-25 [44], even gaseous signal molecule nitric oxide [[45], [46], [47]]. Their findings showed promise in enhancing MSC treatment profiles for various diseases. For example, priming MSCs with TNF- and IFN-γ aided bone regeneration and immune modulation and induced unique microRNA expression in MSCs and their exosomes [18,20]. Additionally, priming MSCs with IFN-γ increased their expression of immunosuppressive molecules such as TGF-, HGF, and PGE2, which mediated their immunosuppressive effect on activated lymphocytes [19,48]. Therefore, we summarize most priming strategies with findings, as shown in (Table 1).

**Table 1:** Highlighted priming strategies and therapeutic applications of primed mesenchymal stem cells.

1st author/type of materials	•PMID	Priming strategies/materials	Molecular mechanism/outcomes
Cytokines			
Agung Putra	• 30,455,748	TNF-α	Improve anti-inflammatory effects through IL-10 and TGF-β.
Moïra François	21934657	IFN-γ & TNF-α	Regulate T-cell proliferation and macrophage polarization
Lei Shen & Aijun Yang	•29,027,451 30016780	IL-8	Enhance cell survival, osteogenesis and chondrogenesis by regulating Akt/STAT3& PI3K/Akt pathways.
Elena Redondo-Castro	•28,412,968	IL-1β	Improve MSCs migration and antiinflammaton effects.
Raghavan C & Marjolijn D	28,713,871&21898680	IFN-γ	Balance of immunosuppressive effects and improve the therapeutic potency of MSCs
Amruta Barhanpurkar-Naik	•28,705,238	IL-3	Modulates MSCs' osteogenesis and chondrogenesis
Tengxiao Ma	•30166501	IL-17	Improvement of the immunoregulatory properties
Ahmed A El-Zayadi	•27,940,584	IL-22	Promotes osteogenic differentiation
Weizi Cheng	•28,979,689	IL-25	Increases immunoregulation roles of MSCs
Bioactive molecules			
Tung-Sheng Chen	35328586	EGCG	Improve the regenerative roles of MSCs in pancreatic tissues
Hamid Yaghooti	•30,639,962	palmitate and astaxanthin	Inhibit IL-6, VEGF, and MCP-1
Sabiha Fatima	•33,488,075	Selenium	Activates MSCs' viability and differentiation by inducing JNK/FOXO3 pathway
Apurva Patel	30548337	Tribulus terrestris saponins	Decreases oxidative stress
Wu Duan	30454697	Rapamycin	Induced expansion of regulatory T cells
Mahmoud Al-Azab	•32,235,006	Indian Hedgehog	Improve antiaging mechanisms of MSCs by inhibiting 4EBP1& p70S6K1/2 phosphorylation
Kamal Hezam	•36,949,464	PGE2	Mediate macrophage polarization and cytokine production.
John Girdlestone	•26,276,002	rapamycin, everolimus, FK506 or cyclosporine	Enhancing immunomodulatory potency of MSCs
Lynda Bourebaba	•30,553,132	Cladophora glomerata methanolic extract	Promotes MSCs' viability and presents anti-inflammatory and immunomodulatory effects.
Growth factors			
Caroline Gorin	26798059	FGF-2	Improve angiogenic effects by regulating HGF and VEGF.
Cong Ma	30501721	TGF-β	Enhance the immunomodulating effects of MSCs/Increase cell survival and rejuvenation
Tatiana Lopatina	30514962	PDGF	Induce antiinflammatory cytokine production like IL-10
Junghyun Park	30409167	bFGF	improves the therapeutic effects of AF-MSC-derived CMs on tissue repair and regeneration.
Hypoxia			
Julie Beegle	•25,702,874	hypoxia (1 % O₂)	Reduced cell death, cytochrome *c* & HO-1 levels, and improved survival in vivo.
Ying-Wei Lan	25986930	hypoxia (1.5 % O₂)	Upregulation of hepatocyte growth factor
Jun Hee Lee	28635661	hypoxia (2 % O₂)	Enhance survival, proliferation angiogenic and cytokine production via HIF-1α-GRP78-Akt
Bailong Li	29183009	hypoxic conditions (2.5 % O2)	Improve their proliferation and antioxidant ability
Zhou Zhilai	27085204	physioxia (5 % O2, P-UCMSC)	Increased migration, survival, tissue and axonal regeneration
Culture conditions			
Zhao Huang	25746258	3D cell culture alginate/chondroitin sulfate	Activate chondrogenesis via initial cell-cell contacts.
Xuebin Chen	23337703	3D cell culture collagen hydrogel scaffold	Improve chondrogenesis differentiation via upregulation collagen II, aggrecan, COMPS.
Qingyang Meng	23337703	3D cell culture affinity peptide sequence (E7) and hydrogel	There was an enhancement in cell longevity, production of matrix, and an improved capability for chondrogenic differentiation.
Xiaoyu Sun	•29,644,091	3D cell culture collagen/hydroxyapatite, hydroxyapatite and biphasic calcium phosphate	The greatest bone-forming potential was demonstrated in collagen/hydroxyapatite, while the least effective was observed in hydroxyapatite.

Most active corresponding authors’ countries on priming mesenchymal stem cells.

**Fig. 2:**
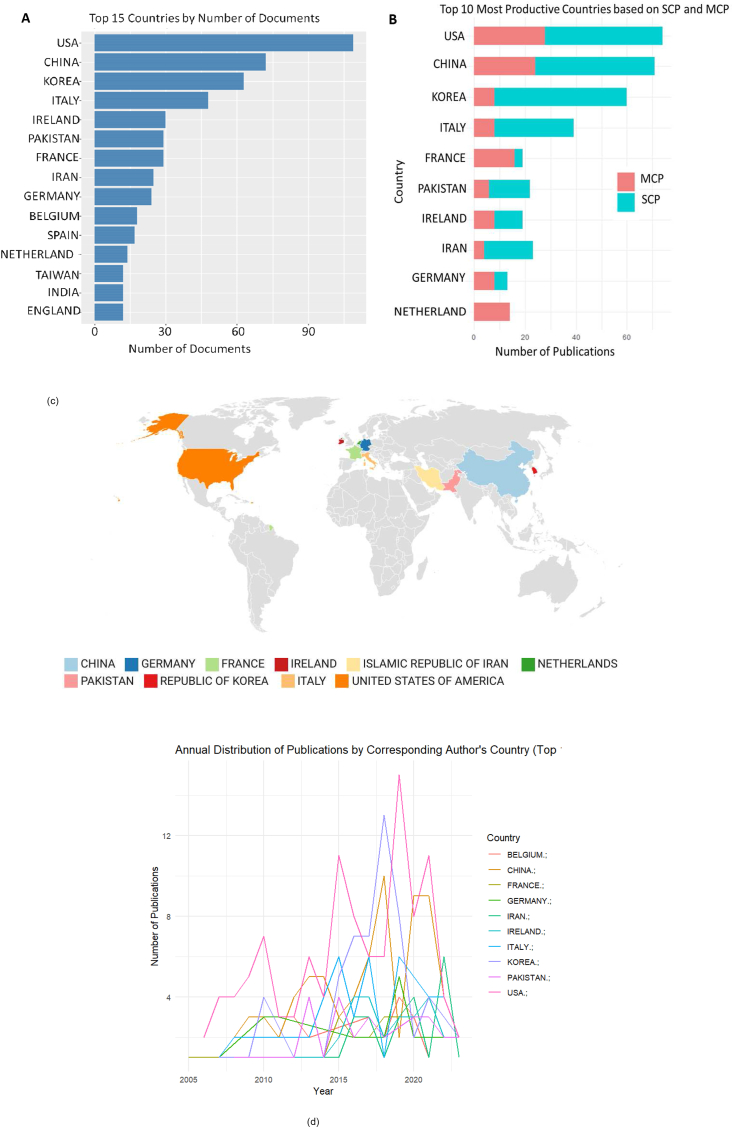
Geographic Contribution to Priming Mesenchymal Stem Cell Research. A. Top 15 countries by publication count. B. Analysis of the most productive countries, indicating Single Country Publications (SCP) and Multiple Country Publications (MCP). C. Geographical distribution map of the productive corresponding authors' countries. D. Time-based distribution of publications by corresponding author's country, illustrating the annual contribution.

In the analyzed collection of documents relating to the priming of mesenchymal stem cells, the United States emerged as the leading contributor with 102 published articles, highlighting its active role in this research area. China followed closely with 95 articles, showing its significant commitment to the field. South Korea marked the third-highest volume of output, contributing 68 publications to the pool of research. Italy followed with a contribution of 47 articles, underscoring the country's persistent endeavors in stem cell research. Other prominent contributors included France with 35 articles, Pakistan with 28 articles, and Iran and Ireland, each contributing 27 articles (Fig. 2A–C). This global distribution of research emphasizes the worldwide interest and collaborative nature of priming mesenchymal stem cell study, with annual trends shown in (Fig. 2D). These geographically diverse contributions offer a variety of perspectives and approaches to this evolving research field. The specific breakdown of publications by each country is elaborated further in (Table 2). Our findings reveal a significant concentration of research activity in the United States and China, underscoring their leadership and substantial investment in this domain. This geographical distribution reflects broader trends in global scientific research, where these countries often lead in biomedical innovations due to their robust research infrastructures, substantial funding mechanisms, and a strong emphasis on advancing medical science. South Korea, Germany, England, and France also emerge as key contributors, illustrating the global interest and collaborative nature of MSC priming research. The active participation of these countries highlights diverse approaches to regenerative medicine and the universal recognition of MSCs' therapeutic potential. This geographical spread not only demonstrates the international effort to harness the benefits of MSC priming for various medical applications but also suggests potential areas for international collaboration to further advance the field. Through this analysis, we aim to emphasize the importance of cross-border partnerships in driving innovation and maximizing the therapeutic applications of MSC priming in regenerative medicine and beyond.

**Table 2:** Corresponding author's countries and total citations per country.

Country	Articles	Freq	SCP	MCP	MCP_R	TC	AAC
USA	102	0.18021	74	28	0.275	4792	46.98
CHINA	95	0.16784	71	24	0.253	3767	39.65
KOREA	68	0.12014	60	8	0.118	2074	30.50
ITALY	47	0.08304	39	8	0.170	2870	61.06
FRANCE	35	0.06184	19	16	0.457	2309	65.97
PAKISTAN	28	0.04947	22	6	0.214	502	17.93
IRAN	27	0.04770	23	4	0.148	511	18.93
IRELAND	27	0.04770	19	8	0.296	1014	37.56
GERMANY	21	0.03710	13	8	0.381	921	43.86
NETHERLANDS	19	0.03357	5	14	0.737	1708	89.89

SCP: Single Country Publications.

MCP: Multiple Country Publications.

MCP-R: MCP_Ratio

AAC: Average Article Citations.

TC: Total Citations.

### 3.2 Most active authors on priming mesenchymal stem cells

**Fig. 3:**
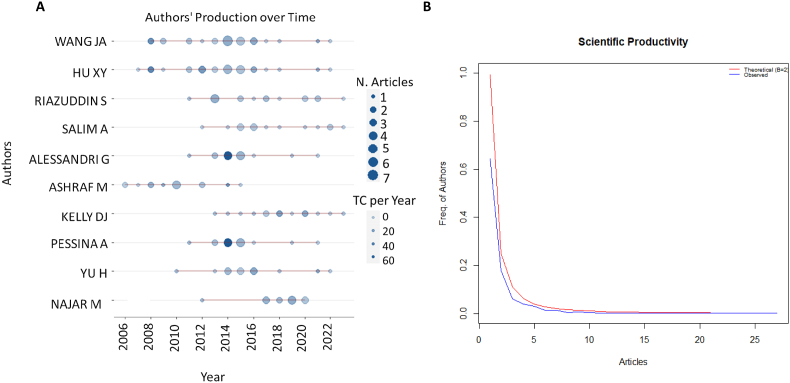
Most active authors on priming mesenchymal stem cells. A. Authors' production over time. B. Estimation of Lotka's law coefficient, demonstrating the publication frequency among authors as an inverse square law—this illustrates that a small number of authors contribute a large portion of publications.

In the field of priming mesenchymal stem cells, a considerable number of 2448 authors have made contributions to the published literature. However, only a group of 20 authors can be recognized for publishing more than 10 articles individually. This analysis offers insights into the prolific contributions and publication patterns within the field (Fig. 3A). highlights the temporal production of key authors, revealing a core group of researchers whose work has significantly advanced MSC priming research. This pattern not only illustrates the central role of these individuals in driving the field forward but also underscores the collaborative nature of this scientific domain. (Fig. 3B), depicting Lotka's law coefficient estimation further emphasizes the distribution of publication productivity among authors. Lotka's law, an important bibliometric principle, suggests that the number of authors making a single contribution to a field far exceeds those making multiple contributions, following an inverse square law distribution [49]. This observation in our study corroborates with Lotka's principle, indicating a small number of highly prolific authors within MSC priming research. The insights derived from these analyses highlight the concentrated impact of a few researchers in shaping the discourse and direction of MSC priming studies. It underscores the importance of recognizing and fostering the contributions of leading scientists while also pointing to the potential for broader engagement and collaboration across the research community to further enrich the field.

### 3.3 Most productive journals on priming mesenchymal stem cells

**Fig. 4:**
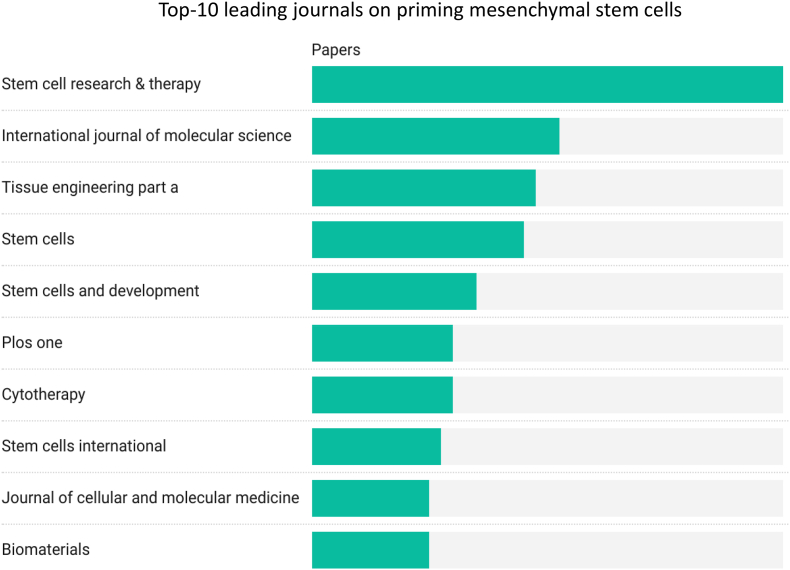
Leading journals on priming mesenchymal stem cells.

The assembled documents come from 228 different sources, including journals, books, and other publication platforms. Among these, only 10 journals have published 10 or more articles pertaining to priming mesenchymal stem cells. The journal that published the majority of these articles was Stem Cell Research & Therapy, contributing 40 articles to the total count. This was followed by the International Journal of Molecular Science, which published 21 articles, and then Tissue Engineering Part A, which published 19 articles. The top 10 journals combined account for 28 % of the analyzed documents, as further detailed in (Table 3 and Fig. 4). This distribution underscores the pivotal role these journals play in disseminating key findings and advancements in MSC priming research. The high citation counts and frequency of publications in these journals not only reflect their importance to the scientific community interested in regenerative medicine and stem cell therapy but also indicate a broader readership and significant impact on the field. Such journals have become primary outlets for groundbreaking research in MSC priming, contributing to the development of therapeutic strategies and enhancing our understanding of stem cell biology. The inclusion of citations per journal in our analysis provides further insight into the influence of these publications, offering a quantitative measure of their contribution to advancing research and knowledge in this specialized area.

**Table 3:** Top 10 leading journals on priming mesenchymal stem cells.

N	Journal	Total papers	Total citations	IF 2022
1	Stem cell research & therapy	40	1213	7.5
2	International journal of molecular science	21	645	5.6
3	Tissue engineering part a	19	418	4.1
4	Stem cells	18	2447	5.2
5	Stem cells and development	14	457	4
6	Plos one	12	873	3.7
7	Cytotherapy	12	180	4.5
8	Stem cells international	11	304	4.3
9	Journal of cellular and molecular medicine	10	377	5.3
10	Biomaterials	10	955	14

### 3.4 Keyword analysis and trend topics on priming mesenchymal stem cells

**Fig. 5:**
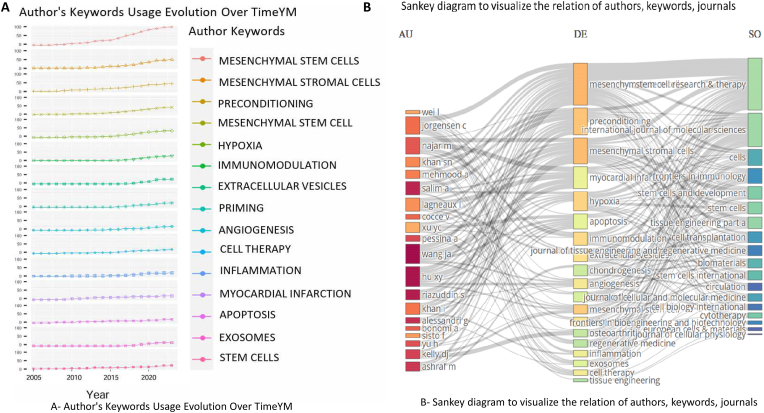
Keyword trends A. Timeline of author keyword usage in priming mesenchymal stem cell research. B. Sankey diagram visualizing the relationships between authors (AU), author keywords (DE), and publication sources (SO).

The examination of keywords and trends was conducted utilizing the Bibliometrix package and VOSViewer software, enabling a comprehensive analysis (Fig. 5A). represents the outcome of this analysis, illustrating the most commonly used author keywords aside from the search keywords. These frequently employed author keywords encompass a range of crucial topics in the field. They included terms such as “Mesenchymal Stem Cells,” “Mesenchymal Stromal Cells,” “Preconditioning,” “Hypoxia,” “Immunomodulation,” “Extracellular Vesicles,” “Priming,” “Angiogenesis,” “Cell Therapy,” “Inflammation,” “Myocardial Infarction,” and “Apoptosis.” These keywords highlight the key themes and areas of focus in research pertaining to priming mesenchymal stem cells. Three fields are plotted to visualize the main items of the three fields (authors, keywords, journals) and how they are related through a Sankey diagram (Fig. 5B). This advanced approach allowed us to generate clearer, more accessible visual representations of the co-occurrence and evolution of keywords over time. Through this analysis, we identified distinct clusters of keywords that signify the main research areas and thematic categories within the field. Over time, the evolution of these keywords reflected shifts in research focus, from basic cellular mechanisms to more applied clinical studies, showcasing the dynamic nature of the field. This analysis provided deeper insights into the conceptual landscape of MSC priming research, underlining the interdisciplinary approaches and the progressive refinement of topics as the field matures. With these visualization tools, we enhanced the clarity of our presentation, making it easier for readers to grasp the complex interactions and trends within the burgeoning field of MSC priming.

### 3.5 Conceptual aspects and coword analysis of priming mesenchymal stem cells

**Fig. 6:**
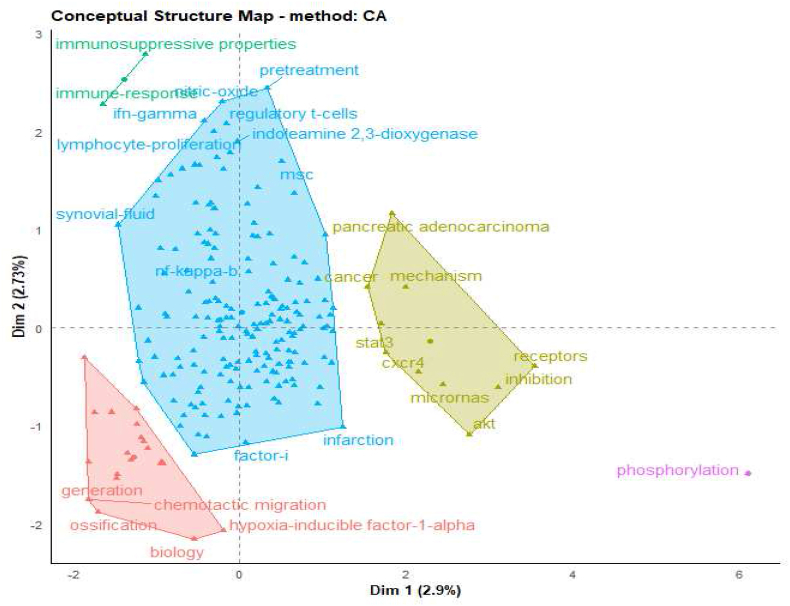
The functional conceptual structure map and keyword clusters of priming mesenchymal stem cell papers.

The inclusion of conceptual aspects and coword analysis in our study of priming mesenchymal stem cells was undertaken to elucidate the intricate framework and thematic connections within this rapidly evolving field. This analytical approach allowed us to dissect the dense network of terminology and concepts, identifying the most central and recurrent themes that define the scope and direction of current research efforts. This analysis involves employing techniques such as multidimensional scaling (MDS), correspondence analysis (CA), or multiple correspondence analysis (MCA) to reduce the dimensionality of the data [50]. By examining the co-occurrence of words within the scholarly literature, we were able to highlight the relationships between various research topics, uncovering patterns of collaboration and intellectual convergence among scientists. In our study, we provide an illustrative example utilizing the function “ConceptualStructure,” which leverages CA or MCA to construct a conceptual framework of the field. We also utilize K-means clustering to identify clusters of documents that share common concepts. The results are then presented on a two-dimensional map, facilitating a clear visualization of the relationships between concepts [[50], [51], [52]]. The “conceptualStructure” function incorporates natural language processing (NLP), including “term extraction” from titles and abstracts. It also uses Porter's stemming algorithm to reduce words to their stem form, improving coword analysis accuracy and offering a deeper understanding of the field's concepts (Fig. 6). This analysis provides valuable insights into the dominant research trends, revealing how certain concepts are interlinked and evolve over time. It offers a visual representation of the conceptual landscape, enabling us to pinpoint emerging areas of interest and potential gaps in the existing body of knowledge. Ultimately, the coword analysis serves as a foundational tool for synthesizing the complex array of research activities in MSC priming, guiding future inquiries, and fostering a more coherent understanding of the field's development and potential research trajectories.

**Fig. 7:**
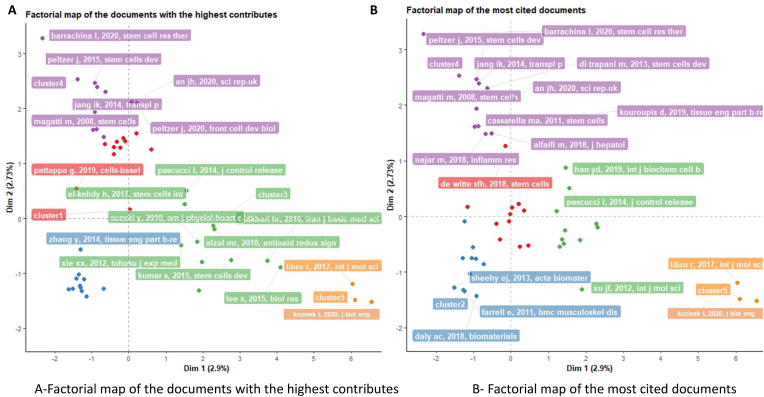
Impactful Documents in MSC Priming Research. A- Factorial map of the documents with the highest contributions among priming mesenchymal stem cell papers. B- Factorial map of the most cited documents among priming mesenchymal stem cell papers.

A factorial map of the documents with the highest contributions is typically a visual representation of the correspondence analysis. It shows the relationships between categories (in this case, documents) as well as their contribution to the analysis. Each document is represented as a point, and the distance between the points indicates how similar or dissimilar the documents are. The documents with the highest contributions (those that contribute most to the variability in the dataset) would likely be prominently displayed on the map. Therefore, the factorial map of the documents with the highest contributions can be interpreted as a visualization of the documents that have the most significant impact on the overall conceptual structure of your bibliographic data frame (Fig. 7A). The conceptual structure (factorial map of the most cited documents) in (Fig. 7B) is a graphical representation that shows how these documents are related based on their citation data. The distances between points reflect the citation similarities (or differences) among the documents, aiding in the identification of similar document clusters and providing an overview of the citation data's structure.

### 3.6 Authors’ keyword visualization on priming mesenchymal stem cell papers

**Fig. 8:**
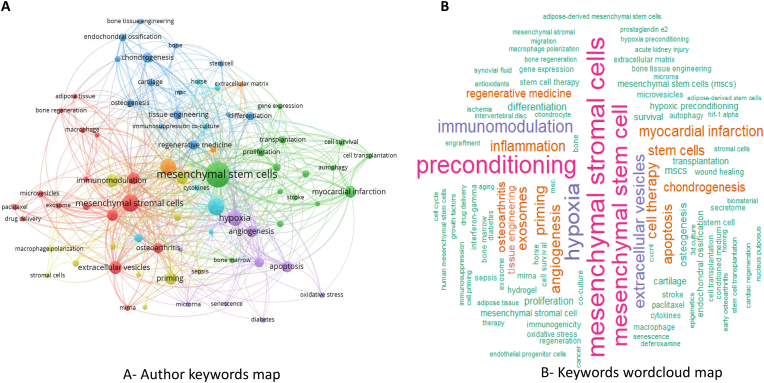
Keywords visualization on priming mesenchymal stem cell papers. A. Author keywords co-occurrence network visualization. B. Wordcloud visualization analysis.

Before an author submits a manuscript to a journal, they must provide keywords that accurately encapsulate the primary concepts put forth in their work. Analyzing these keywords serves as a means of uncovering pivotal areas of research. In this analysis, the term ‘occurrence’ refers to the frequency with which a keyword appears across articles, whereas ‘co-occurrence’ denotes the frequency with which a keyword appears in tandem with others. More accurately reflecting research trends, keywords that frequently co-occur can reveal hotspots in the research. A baseline frequency of 10 occurrences was established for author-provided keywords. Of all the keywords evaluated, only 62 surpassed this threshold, which was then organized into 8 clusters. The most commonly used keywords provided by authors were ‘mesenchymal stem cells' and ‘preconditioning’, with occurrence frequencies of 108 and 68, respectively. These were followed by ‘hypoxia’ and’ mesenchymal stromal cells', with frequencies of 67 and 59, respectively. The total strength of connections between keywords, as depicted in (Fig. 8), hinges on the number of documents in which the pair of keywords appear together, illustrating the relationship between specific research areas. The thickness of a connection between two keywords directly correlates with the strength of their relationship within the network. The thickness of the lines between keywords reflects the strength of their relationship, with closely interconnected keywords sharing the same color, indicating their commonality.

### 3.7 Cocitation visualization map on priming mesenchymal stem cell papers

**Fig. 9:**
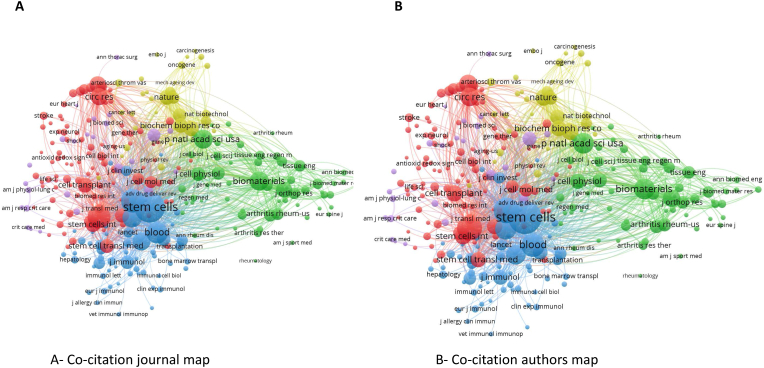
Cited sources visualization on priming mesenchymal stem cell papers.

A multitude of studies underscore the significance of a journal within any scientific discipline. This analysis aids authors in publishing their work in reputable journals and helps readers find the most relevant sources for their research. The gathered data were charted for the visualization of cited sources, as demonstrated in (Fig. 9). The minimum citation count for any given source was fixed at five. Among the sources assessed, only 27 surpassed this threshold, consequently forming four clusters. The journal ‘Stem Cell Research & Therapy’ took the lead with a total link strength (TLS) of 89. It was followed by ‘Stem Cells' (TLS = 59), ‘International Journal of Molecular Science’ (TLS = 52), and ‘Stem Cells and Development’ (TLS = 41) (Fig. 9).

Coauthorship country's visualization on priming mesenchymal stem cell papers.

**Fig. 10:**
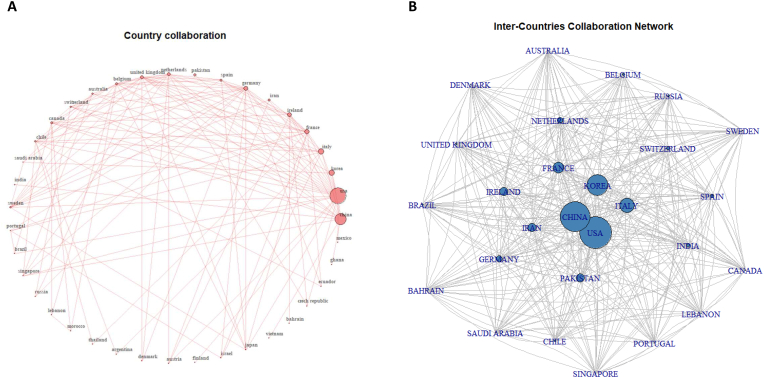
Coauthorship Analysis by Country in MSC Priming Research. A–B: Coauthorship country visualization of priming mesenchymal stem cell papers.

As illustrated in (Fig. 5), the various countries and regions contributing to research related to mesenchymal stem cell priming over time were mapped for overlay visualization. A minimum document count of 41 was set for each participating country or region. Out of the total countries or regions, only 27 made the cut and were subsequently displayed. The evaluation metrics were total link strength, used as the weight, and the average number of publications per year, which served as the scores. The countries boasting the highest total link strength were the USA, China, Germany, England, and France, with respective scores of 94, 41, 37, 34, and 34 (Fig. 10).

### 3.8 Bibliographic coupling map

**Fig. 11:**
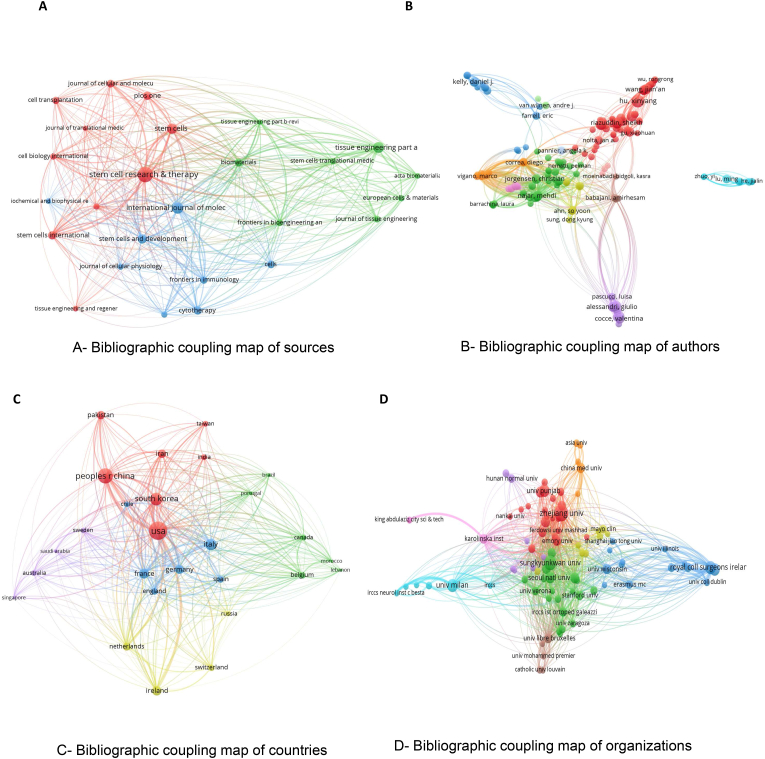
Bibliographic Coupling in MSC Priming Research. A-D. Bibliographic coupling maps for sources, authors, countries, and organizations, highlighting connections based on shared references.

In this section, we dissect various components involved in priming MSCs, encompassing sources, authors, institutions, and countries (Fig. 11). presents the bibliographic coupling maps for these components, each having a minimum citation count of five. Bibliographic coupling serves as a tool for identifying connections between documents, implying that they have a common set of references and thereby revealing unity within the research field. As such, it ranks sources, authors, institutions, and countries or regions based on total link strength. TLS is a metric that evaluates the robustness of a scholar or a paper's connections with others in the same field. It quantitatively depicts the level of interaction and cooperation among scholars, institutions, and areas. This metric depends on the number of thresholds selected for assessment. Presently, the coupling strengths among sources are mainly focused on the subject of priming mesenchymal stem cells. This implies that collaborations and intellectual links between these entities are predominantly centered on this particular research topic. This focus serves to highlight the mutual research interests and the profound interconnectedness among these components in the realm of primed MSCs.

## 4 Discussion

The results of these analyses underline key trends, contributing factors, and hotspots within the field of mesenchymal stem cell priming research. The research analysis provided presents several points of interest regarding the development and current state of priming mesenchymal stem cells within the scientific literature. The number of published documents on priming MSCs has grown significantly since 2003, highlighting the growing attention and interest in this particular field. The marked growth in research productivity, peaking in 2019 and closely followed by 2020, speaks to the rising significance of MSC priming in regenerative medicine and its potential for therapeutic applications. However, there is a modest decrease in publication count observed in 2023, which may reflect a stabilization or temporary lull in research output. A slight decrease in publications in 2023 suggests a stabilization or temporary slowdown in research output, but numbers may rise by year's end. This bibliometric analysis on priming mesenchymal stem cells is not just a mere enumeration of publications and identification of trends; it provides a scaffold for the scientific narrative that has shaped this field over two decades. By dissecting the growth patterns, seminal works, and pivotal shifts in research focus, we offer a nuanced understanding of how MSC priming has evolved from a niche interest to a cornerstone of regenerative medicine research. This historical perspective enriches the reader's appreciation of the field's development and the collective effort behind its advancements.

Mesenchymal stem cells are of significant interest in the field of regenerative medicine, capable of transforming into various cell types and regulating immune responses. In various preclinical and clinical trials for an extensive range of diseases, including neurodegenerative diseases, cardiovascular disorders, and autoimmune diseases, MSCs have demonstrated their therapeutic potential [2,53]. Several strategies have been used for priming MSCs to improve their therapeutic efficacy. By modulating the cellular environment with various biofactors and chemical factors, researchers have found promising ways to enhance MSC treatment profiles for various diseases. Priming MSCs with TNF-α and IFN-γ, for instance, has been shown to aid bone regeneration and immune modulation [54]. Priming MSCs with hepatocyte growth factor-expressing MSCs (HGF-eMSCs) has been shown to enhance their therapeutic potential for cardiac repair [55]. Moreover, MSCs primed with specific cytokines demonstrated improved immunoregulatory activity, which could potentially be beneficial in reducing disease severity [10]. Therefore, this work was designed to highlight a trend analysis of the literature in the field of priming MSCs.

The keyword and trend topic analyses revealed a set of terms frequently employed by authors, underscoring the breadth and diversity of topics within the field. Notably, the most recurrent keywords were “Mesenchymal Stem Cells,” “Mesenchymal Stromal Cells,” “Preconditioning,” and “Hypoxia,” which suggest that these terms represent the focal points of the current research in this field. The coword analysis provided a nuanced understanding of the conceptual framework of MSC priming research. By using multidimensional scaling, correspondence analysis, or multiple correspondence analysis, we distilled the high-dimensional data into an interpretable two-dimensional map, revealing the key relationships and interactions among various research concepts. These techniques, aligned with the guidelines from the relevant literature [23], were crucial in helping us navigate the complex landscape of this field. Through the visualization of author keywords, we discerned the current trends and research interests in the domain of MSC priming. The connections between the terms “mesenchymal stem cells” and “preconditioning” were particularly strong, suggesting that these two areas might share something common in terms of research approaches or underlying biological mechanisms. This observation complements the insights provided by the cited source visualization, which indicated that the majority of the leading sources of information on this topic are specialized stem cell journals.

Our overlay visualization of the countries and regions participating in MSC priming research demonstrated that the USA, China, Germany, England, and France are at the forefront of this field. The total link strength of these countries indicates their significant contribution to MSC priming research, perhaps reflecting the robust scientific infrastructure and resources available in these nations. This finding underscores the need for continued international collaboration in this research domain, as suggested by the literature on international cooperation on migration [56]. The global distribution of contributions to MSC priming research, as revealed through our analysis, underscores the importance of collaboration across borders. By showcasing the contributions from various countries, we not only show the international nature of the scientific endeavor but also advocate for increased collaboration. This highlights the added value of diversity in perspectives and expertise, encouraging researchers to seek cross-cultural partnerships that can enhance the quality and impact of their work.

While this study offers an insightful bibliometric analysis of the research on priming mesenchymal stem cells, several limitations should be noted. First, the study only included English-language documents, potentially missing relevant research published in other languages. Second, the study's reliance on specific databases could have resulted in the exclusion of important research published elsewhere. Third, the keyword-based search could have overlooked key documents that used different terminology. Fourth, the temporal coverage of the study, ending in mid-2023, may not account for more recent developments. Fifth, the national contribution was determined based on the country of the corresponding author, which may not accurately represent the extent of international collaborations or where the research was conducted. Last, both coword analysis and citation analysis come with limitations due to the reliance on specific keywords and citation counts, potentially skewing the interpretation of popular research themes and influential works in the field. These limitations should be considered when interpreting the findings and should be addressed in future bibliometric analyses in the field of priming mesenchymal stem cells.

## 5 Conclusion

The trend analysis of the literature demonstrates the robust growth and increased interest in the field of priming MSCs since 2003. Multiple strategies to enhance the therapeutic potential of stem cells have been investigated, and many have shown promising results in the treatment of various diseases. The majority of contributions have come from the United States and China, highlighting their significant role in the global research landscape. The most productive journal in this domain, Stem Cell Research & Therapy, underscores the importance of specialized journals as platforms for disseminating knowledge in this field. Keyword and coword analyses illuminated the central research topics and provided insights into potential avenues for future investigations. With ongoing research efforts and promising findings, it is expected that the field of MSC priming will continue to expand, facilitating advances in stem cell-based therapies.

## Funding

This study was financially supported by open funding from the 10.13039/501100004945Nankai University Eye Institute (NKYKD202203), the Research Project on Skin Injury & Repair (BKJ21J016, 2021XB008), and the 10.13039/501100006606Tianjin Natural Science Foundation (22JCZXJC00170, 21JCZDJC00070).

## Availability of data and materials

The original contributions presented in the study are included in the article, and further inquiries can be directed to the corresponding author.

## Competitive interests

The authors have declared that they have no competing interests.

## Ethics approval and consent to participate

The study did not involve the direct recruitment of human participants or the use of animals. No ethical approval was required.

## CRediT authorship contribution statement

**Kamal Hezam:** Writing – original draft, Data curation. **Enze Fu:** Writing – original draft, Validation, Data curation. **Jun Zhang:** Writing – review & editing, Visualization, Validation, Conceptualization. **Zongjin Li:** Writing – review & editing, Writing – original draft, Supervision, Resources, Project administration, Funding acquisition.

## Declaration of competing interest

None

## Data Availability

No data was used for the research described in the article.
